# Microbiological contamination of ear, nose and throat (ENT) units

**DOI:** 10.3205/dgkh000319

**Published:** 2019-02-01

**Authors:** Marco Krull, Joerg Steinmann, Evelyn Heintschel von Heinegg, Jan Buer, Anke Sucharski, Stefan Mattheis, Stephan Lang, Birgit Ross

**Affiliations:** 1 Universitätsmedizin Essen, Krankenhaushygiene, Essen, Germany; 2 Universitätsmedizin Essen, Institut für Medizinische Mikrobiologie, Essen, Germany; 3 Institut für Klinikhygiene, Medizinische Mikrobiologie und Klinische Infektiologie, Paracelsus Medizinische Privatuniversität, Klinikum Nürnberg, Germany; 4 Universitätsmedizin Essen, Klinik für Hals-Nasen-Ohren-Heilkunde, Essen, Germany

**Keywords:** hospital hygiene, ENT treatment units, microbial contamination

## Abstract

**Aim:** In ENT (Ear, Nose and Throat) treatment units, medical devices for examination are commonly stored on open trays. The aim of this study is to investigate whether open storage is a relevant cause for microbiological contamination of ENT instruments during a working day.

**Methods:** Qualitative and quantitative tests, such as imprints and swabs, were performed on the instruments and the surfaces of the treatment units in an ENT outpatient clinic at the beginning and at the end of consultation hours. The microbiological analysis of the samples focused on potential pathogens, e.g., *Staphylococcus aureus* or *Pseudomonas aeruginosa*, bacteria of skin and oral microbiota, as well as the number of colony forming units (CFU). The samples were collected at three distinct ENT treatment units over five working days.

**Results:** The samples taken at the beginning of consultation hours showed a low number of CFU and no pathogens. Overall, 5% of the instruments were contaminated with bacteria of skin microbiota. At the end of a working day, this rate increased significantly to 17.5% (p<0.01). At the beginning of the working day, the mean number on the instrument trays was 4 CFU/25 cm², which increased to 34 CFU/25 cm² at the end of the working day. In some cases of the imprints taken at the end of the working day showed that a bacterial lawn had formed. In two cases, the pathogens *Ralstonia picketii* and *Enterobacter cloacae* were detected; in another case *Bacillus spp*. was identified. The contamination of ENT instruments and the ENT treatment unit increased significantly (p<0.01) over the duration of consultation hours.

**Conclusion:** The results show that the current hygiene requirements for storage und reprocessing are not sufficient to conform to the mandatory guidelines of the German Commission on Hospital Hygiene and Infection Prevention. Although we could not find a pressing risk for the patients, we also cannot exclude it in the long term. Thus, new concepts are needed.

## Zusammenfassung

**Ziel:** Die HNO-Behandlungseinheit ist der zentrale Arbeitsplatz des HNO-Arztes. Auf dieser werden die benötigten Untersuchungsinstrumente in der Regel offen gelagert. Diese Untersuchung soll der Frage nachgehen, ob es im Laufe eines Arbeitstags zu einer relevanten mikrobiellen Kontamination des HNO-Instrumentariums kommt.

**Methoden:** In einer HNO-Ambulanz wurden vor Beginn und nach Ende der Sprechstunde Abstrichuntersuchungen der Instrumente und Kontaktkulturuntersuchungen der Instrumentenablageflächen vorgenommen. Die mikrobiologische Untersuchung erfolgte auf potenziell pathogene Mikroorganismen (z.B. *Staphylococcus aureus*, *Pseudomonas aeruginosa*), Vertreter der Haut- und Rachenflora sowie auf die Gesamtkoloniezahl. Es wurden an insgesamt 5 verschiedenen Arbeitstagen an jeweils 3 unterschiedlichen HNO-Einheiten Umgebungsuntersuchungen durchgeführt.

**Ergebnisse:** Vor Arbeitsbeginn wurden geringe Gesamtkoloniezahlen und keine potenziell pathogenen Bakterien gefunden; insgesamt waren 5% der Instrumente mit Vertretern der Hautflora kontaminiert. Am Ende des Ambulanzarbeitstags stieg diese Rate auf 17,5% an (p<0,01). Zu Beginn des Arbeitstags lag die durchschnittliche Kontaminationsrate der Instrumentenablagen bei 4 KBE/25 cm², was auf 34 KBE/25 cm² zum Ende des Arbeitstages anstieg. Auf einigen der am Ende des Tages entnommenen Kontaktproben zeigte sich bakterielles Rasenwachstum. In zwei Fällen gelang der Nachweis von Pathogenen (*Ralstonia picketii*, *Enterobacter cloacae*), in einem weiteren Fall der Nachweis von *Bacillus spp*. Im Laufe eines Arbeitstages nahm sowohl die Kontamination der HNO-Instrumente als auch der HNO-Einheit stark zu.

**Schlussfolgerung:** Die Ergebnisse verdeutlichen, dass die derzeitigen Aufbereitungsvorschriften für HNO-Einheiten nicht den Vorgaben der Kommission für Krankenhaushygiene und Infektionsprävention entsprechen. Es müssen neue Konzepte entwickelt werden.

## Introduction

The central place of work for an ENT (Ear, Nose and Throat) physician is the ENT unit. This unit contains various examination instruments and medical devices for flushing and aspirating. In contrast to dental units, all clean instruments are stored uncovered in the ENT unit. Once used on a patient, instruments are placed in a separate box; they are reprocessed after usage or at the end of the day (Guideline of the German Society of Hospital Hygiene [1], recommendation of local health authority Frankfurt, Germany [2]) or after one week (recommendation of Atmos^©^, manufacturer of ENT units). 

During an ENT examination process, usually four to six different instruments are necessary. This means that the physician has to take out each clean instrument individually during the workflow. This practice may lead to contamination of clean instruments that will be used on a different patient.

Many of the medical devices used by an ENT physician during an examination are classified as either semi-critical A or semi-critical B [3]. Medical devices are categorized as “semi-critical” if they come in contact with non-intact skin or mucosa. Category A devices can be effectively cleaned and safely reprocessed; category B devices have lumens or rough surfaces, reprocessing may affect functional use, or have a limited number of uses. Thus, category B devices have more stringent requirements for reprocessing. According to KRINKO (Commission on Hospital Hygiene and Infection Prevention at the Robert Koch Institute, Germany), this requires, among other things, mechanical reprocessing.

Reprocessed “semi-critical” devices have to be stored in a manner that excludes recontamination [3]. However, this is not the case in current ENT units that use uncovered storage. Patients may cough or vomit during an examination; this is a potential risk of contamination for instruments and surfaces of the unit. This is a particular concern for patients with tracheostomy, due to an increased rate of colonization with pathogens, including multiresistant organisms [4]. The contamination risk may also be increased due to the fact that patients with respiratory infections are often treated by an ENT physician. Thus, precipitation of bacteria in aerosols from the airway is not unlikely.

To the best of our knowledge, there are no studies which analyzed the microbiological contamination of ENT medical devices; the only available data are from dental units [5], [6], [7], [8]. These and similar data contributed to changes in the workflow management for dentists: there are either standardized sets with instruments or the instruments are taken from the storage area when they are needed [9]. This practice definitely leads to higher patient safety; however, its disadvantages are higher costs and consumption of resources.

Our study addresses the question of whether microbiological contamination in an ENT unit during a normal working day might pose a risk for patient safety. Thus, contamination must be analyzed qualitatively and quantitatively.

## Methods

### Setting

The study was performed in the ENT outpatient clinic of the University Hospital Essen. Each working day, approximately 70 patients are treated in five different treatment rooms. The ENT work stations are produced by Atmos^©^ (Lenzkirch, Germany). The instruments are delivered sterilized but are not stored under sterile conditions. The reprocessing procedures are performed by the central sterile supply department of the University Hospital Essen. After use on a patient, the device is placed in a separate box for disposal. At the end of the working day, the ENT unit is cleaned completely and all residual instruments are reprocessed. The unit itself and the trays are disinfected with a surface disinfectant containing quaternary ammonium compound (Incidin plus^©^, Ecolab, Monheim, Germany). The next morning, the unit is restocked with sterilized instruments.

### Environmental sampling

**Figure 1:**
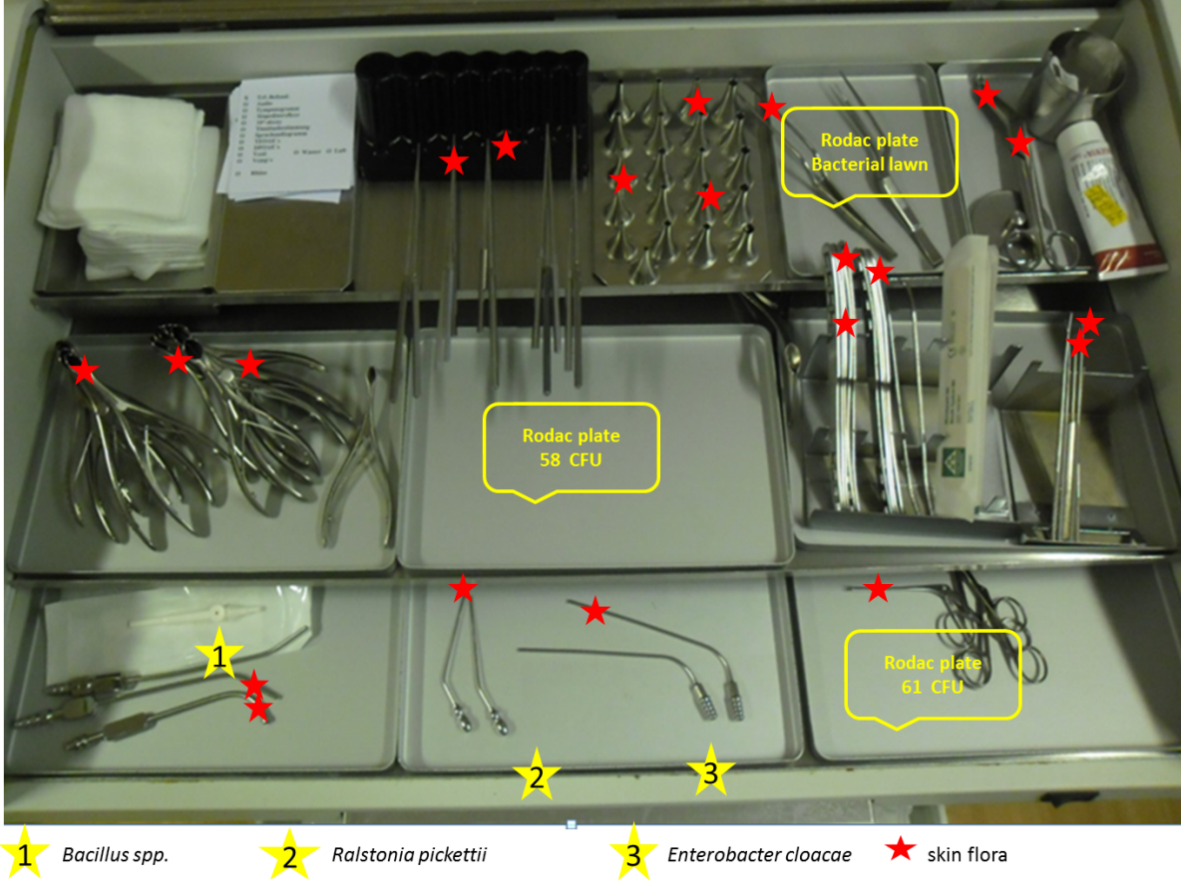
Cumulative representation of bacterial contamination as well as its detection sites on the ENT unit (three examples of increased total bacterial count are displayed with yellow stars)

Before the beginning of the working day in each treatment unit, 13 swabs were taken from the stored disinfected instruments (Port-A-Cul^©^swab) to obtain baseline data regarding contamination. Only the surfaces of the instruments which might have contact with mucosa were sampled (“semicritical instruments”); the handles of the instruments were not swabbed. Also only unused instruments were sampled. Furthermore, different parts of the worktop were swabbed (see Figure 1 (Fig. 1)). The swabs were analyzed for microbiological contamination, especially skin flora, oral flora, pathogens, and spore-formers. To evaluate bioburden, environmental samples were taken (RODAC^©^ plates 25 cm^2^) at 6 determined sites. The samples were analyzed using standard methods at the hygiene laboratory at the University Hospital Essen [10], [11].

### Statistics

Statistical analyses were performed with EXCEL 2013 (Microsoft, USA); figures were prepared with GraphPad Prism (La Jolla, USA). A value of p<0.05 was considered significant, p<0.01 as highly significant and p<0.001 very highly significant.

## Results

In total, 305 swabs from the instruments were taken on five different days; 117 were taken at the beginning of a working day, 188 were taken at the end of the day. Each day, three different ENT units were analyzed. At the beginning of the day, 5.9% (7 of 117) of the instruments were microbiologically contaminated. This contamination always consisted of skin flora. Analyzed in detail, it can be stated that six of these seven contaminated instruments belonged to one single working day. 

**Figure 2:**
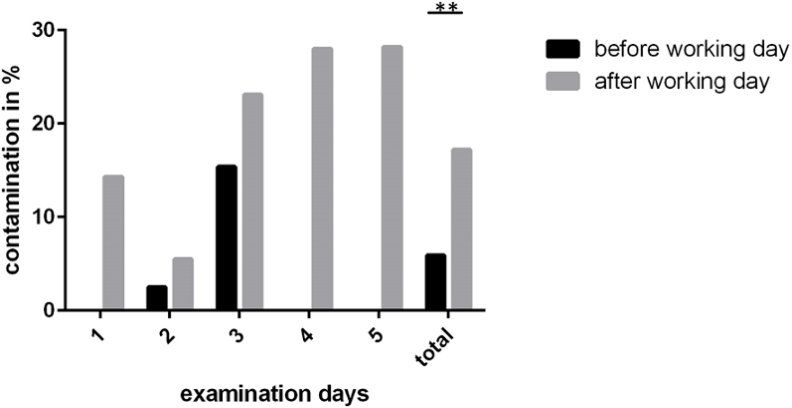
Rate of contaminated instruments before the beginning and at the end of a working day for five consecutive days

At the end of the working day, 17.0% (32 of 188) of the unused instruments were contaminated with bacteria. This increase is significant (p<0.01). In most of the cases the contamination was due to skin flora; however, in 1.6% (3 of 188), the contamination was due to aerobic spore-forming bacteria. The contamination was approximately equally distributed among all investigated ENT units. Figure 2 (Fig. 2) shows the contamination rates on each day.

Furthermore, we performed qualitative analysis of the trays and the ENT unit itself. Besides the above mentioned finding of skin flora, we detected aerobic spore-forming bacteria and, in two cases, pathogens. On the workstation, we found *Ralstonia picketii* and *Enterobacter cloacae*. The places of detection are displayed in Figure 1 (Fig. 1).

**Figure 3:**
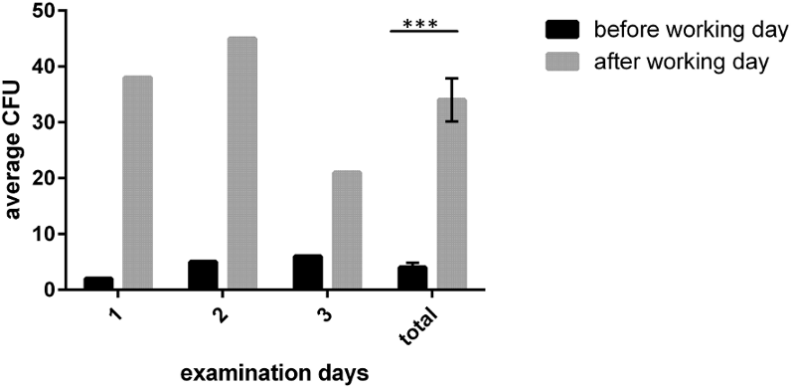
Average contamination (CFU/25 cm^2^) and total CFU on trays used for instruments (increase p<0.001)

For quantitative evaluation of microbial contamination, we performed imprints. In this analysis, 108 samples were taken from trays and surfaces. Of these, 54 samples were taken at the beginning and 54 at the end of the working day. At each time, 6 samples were taken at predefined points. The average contamination at the beginning of the working day was 4 CFU per 25 cm² with a median of 3 CFU/25 cm². At the end of the working day the average contamination was 34 CFU/25 cm² with a median of 29 CFU/25 cm². This increase was significant (p<0.001). Furthermore, in the morning, no sample was contaminated with more than 30 CFU/cm²; in the afternoon, this was the case in more than 22 (of 54) samples. In one case, there were more than 100 CFU/cm²; in two cases there was a bacterial lawn. The results are summarized in Figure 3 (Fig. 3).

## Discussion

Most of the instruments used for ENT examinations have contact with mucosa, e.g. tongue spatula, mirrors, nose spatula. According to the German guidelines for reprocessing, which are mandatory according to law [12], [13], the ENT instruments must be classified as semi-critical [3]. This designation means that these medical devices must be disinfected after use. In addition, contamination of the reprocessed instruments must be prevented, but this might be difficult to achieve. 

Several studies have already dealt with hygiene in ENT facilities and reprocessing of ENT instruments [14], [15], [16], [17], [18], [19], [20]. The current study is the first to evaluate contamination of ENT units or ENT instruments during a working day. 

The study has shown that no pathogens or pharyngeal flora were detectable on these medical devices. As described in the methods section, the ENT unit is stocked with instruments by the nursing staff, after the equipment was sterilized by the central sterile supply department. This, of course, can lead to slight bacterial contamination despite performing hand disinfection. The instruments in the ENT unit classified as “semi-critical” were found nearly completely free of bacteria in the morning. Only in one case were 5% of the instruments already contaminated. This was found in one single ENT unit on one single day. It is possible that the nurse performed insufficient hand hygiene during stocking of the ENT unit on that day. 

During the working day, the rate of contaminated unused instruments increased significantly up to 17%. In this study, only bacteria belonging to the skin flora were detected. This means that the contamination of the instruments is most likely due to the hands of the ENT physician. The physician repeatedly takes instruments from the unit during examination of the patient. Pathogens were found twice; the bacteria were *Ralstonia pickettii* and *Enterobacter cloacae*. Both pathogens are Gram-negative bacteria and known causative agents of nosocomial infections, for example, sepsis or meningitis (*Ralstonia pickettii*), wound infections or urinary tract infections (*Enterobacter cloacae*). Thus, the presence of these bacteria poses a risk to the patients. The pathogens were detected in the ENT unit. Since all cabins are used by all physicians, we suspect a general problem which is not limited to a specific person. Incorrect hand hygiene seems to be the most likely cause. Thus, it is highly probable that instruments can and will be contaminated by the hands of the physician.

When reviewing the total bacterial count, it is still difficult to evaluate the results because there are no generally accepted limits for the contamination of surfaces in hospitals. Basically, bacterial skin flora can be acceptable on semi-critical instruments, e.g., endoscopes [3]. 

In the current study, we examined instruments used on mucous membranes. Thus, we see similarities to the standard in food hygiene [21]. In this standard, contamination of 30 CFU/25 cm² is classified as highly contaminated, which is not acceptable for a commercial kitchen. Studies which examine hand-contact surfaces (e.g., handles, switches, stethoscopes, waste bins, telephones, soft furniture and so on) in hospitals describe 65 CFU/cm² or even 125 CFU/cm² as acceptable [22].

Comparing our data with the criteria of the studies mentioned above [3], [21], [22], it is clear that at the beginning of the working day all surfaces (instruments and trays) fulfill the criteria. At the end of the working day, half of the trays were contaminated at a higher level. In a commercial kitchen, this level would not be acceptable [21]. In some cases, contamination was even higher than one would accept for hand-contact surfaces in hospitals. In our opinion, higher standards for semi-critical instruments should be applied than for hand-contact surfaces, because a higher microbial burden may increase the risk of exposure to pathogens.

In principle, this contamination could be avoided with correct hand hygiene. The work flow during an ENT examination has to be interrupted by hand hygiene every time after touching the patient. The physician should perform hand disinfection for 15 seconds before taking an instrument from the clean tray [23], [24]. A problem is that no disinfectant dispensers from the manufacturer are provided in the treatment units. In addition, there is often not enough space to set up a pump dispenser. However, using a wall dispenser will lead to additional disruptions of the workflow, which might promote noncompliance with hygienic hand disinfection.

With regard to the requirements in the dental field, the question arises whether it may be necessary to transfer these to ENT. We estimate the risk of infection in the field of dentistry to be significantly higher for two reasons:

1. Dental procedures are often associated with injuries of the mucous membranes and bleeding, which is usually not the case in the ENT examination;
2. The contamination risk in the dental sector is significantly increased, as the treatments are often associated with the formation of aerosols [5].

Our study has some limitations. First, we only took samples from small surfaces. The swabbed surface from an instrument is very small, only a few cm² which may lead to a sampling error. This may be the reason why we did not find major contamination on instruments. Furthermore, we did not analyze for viruses, although viruses causing upper respiratory infections remain infectious on surfaces (e.g., about 24 hours for rhinoviruses ) [25]. Taking into consideration that patients with respiratory infections are common in ENT outpatient clinics, there is a potential risk of a viral infection due to the open storage.

In our study, the instruments were reprocessed at the latest at the end of the day and the surfaces were disinfected. This is also recommended by the German Society for Hospital Hygiene [1]. However, in many units, it is common to use instruments for seven days before reprocessing. This procedure is not acceptable, as shown by our data. Furthermore, the replacement of instruments every 24 hours is associated with risks. Thus, we conclude that the mandatory guidelines of the Commission of Hospital Hygiene and Infection Prevention at the Robert Koch Institute in Germany (KRINKO) and the Medical Devices Act for semi-critical medical devices are not being followed. It should be noted that every patient has to be examined with properly reprocessed instruments. Although no acute risk to patients were found in this study, we cannot rule it out in the long term. Thus, new concepts are needed. 

## Notes

### Competing interests

The authors declare that they have no competing interests.
